# Advancing the sexual and reproductive health and human rights of women living with HIV

**DOI:** 10.7448/IAS.18.6.20760

**Published:** 2015-12-01

**Authors:** Mona Loutfy, Rajat Khosla, Manjulaa Narasimhan

**Affiliations:** 1 Women's College Research Institute, Women's College Hospital, University of Toronto, Toronto, Ontario, Canada; 2 Department of Reproductive Health and Research, World Health Organization, Geneva, Switzerland

**Keywords:** HIV, women's health, sexual health, reproductive health, reproductive rights, gender, equity, human rights

## Abstract

**Introduction:**

Many women living with HIV can have safe, healthy and satisfying sexual and reproductive health, but there is still a long way to go for this to be a reality, especially for the most vulnerable amongst them who face repeated violations of their rights.

**Discussion:**

The contributions in this Supplement from researchers, clinicians, programme managers, policy makers, and women living with HIV demands an important appreciation that the field of sexual and reproductive health and human rights for women living with HIV is complex on many levels, and women living with HIV form a very diverse community.

**Conclusions:**

The manuscripts emphasize that attention must be paid to the following critical dimensions: 1) Placing human rights and gender equality at the centre of a comprehensive approach to health programming, in particular in relation to sexuality and sexual health; 2) Ensuring health systems responsiveness to minimizing inequalities in access to health care and quality of care that often do not meet the needs of women living with HIV; 3) Engaging and empowering women living with HIV in the development of policies and programmes that affect them; and 4) Strengthening monitoring, evaluation and accountability procedures to provide good quality data and ensuring remedies for violations of health and human rights of women living with HIV.

## Introduction

An integrated approach to health and human rights lies at the heart of ensuring dignity and well-being of individuals around the world and is linked to improvements in the uptake of services and incidence of positive outcomes. Through the roll out of antiretroviral treatment, advances in overcoming stigma and discrimination, and development of HIV prevention interventions, the HIV and AIDS response has given hope for a healthy life for many around the world. However, for those who remain the most vulnerable, there is not nearly enough progress. Women and girls, for instance, remain especially vulnerable to HIV infection because of a host of biological, social, cultural and economic reasons, including women's entrenched social and economic inequality within sexual relationships and marriage. HIV is not only driven by gender inequality, but it also entrenches gender inequality, leaving women more vulnerable to its impact [1]. Moreover, women and girls at risk of, or living with, HIV have additional challenges linked to sexual and reproductive health that includes risk of unintended pregnancy, complications arising from unsafe abortions and a host of other sexual and reproductive health morbidities. Violence, whether it be physical, sexual and/or emotional, or fear of violence can prevent women from negotiating safer sex and from learning and/or sharing their HIV status if the results turn out positive. In addition, women living with HIV are sometimes blamed for bringing HIV into the family and for being immoral and breaking sexual norms. Many women living with HIV can achieve safe and satisfying sex lives, but there is still a long way to go for this to be a reality for the most vulnerable amongst them who face repeated violations of their rights.

For this special Supplement, we sought for seminal, peer-reviewed contributions that discussed varied perspectives and topics related to sexual and reproductive health and human rights of women living with HIV. These perspectives include contributions from researchers, clinicians, programme managers, policy makers and women living with HIV. The latter perspective is important in allowing this Supplement to hear the voices of the women that we aim to support. The topics in this Supplement are equally varied from HIV pregnancy programming and sexual health to safer disclosure of HIV, mental health and violence, amongst others. This wide range of topics demands an appreciation of the fact that the field of sexual and reproductive health and human rights for women living with HIV is complex on many levels, and women living with HIV form a very diverse community.

The potential solutions regarding gender inequalities [2] and the challenges of ensuring human rights considerations as present in normative bodies [3], policies and programmes [4] reflect two cross-cutting issues, gender equality and human rights, that permeate the whole Supplement and form the foundation for strengthened services that meet the needs of women living with HIV.

The papers on sexual health for women living with HIV were purposively positioned before those on reproductive health, given that sexual health and the right to a safe and satisfying sex life [5] is a topic often not addressed by clinicians despite its vital importance, at a personal level for women, especially in an era of over-criminalization of HIV. Whether in resource-constrained or wealthier settings, women living with HIV should be offered choices and health interventions that would allow them to lead healthier lives. This is true, for instance, of cervical cancer, which is a disease that is preventable, but for which screening and prevention in low-income countries [6] remains a challenge. Two other topics that have developed a significant amount of momentum globally over the past decade include the development and delivery of preconception services for women and couples affected by HIV in resource-constrained settings [7] and the roll out of Option B+ for the management of women living with HIV during the perinatal period [8], which has been a breakthrough for infant, maternal and women's treatment of HIV at the global level, but which needs to be implemented within a rights-based framework.

The next set of papers are about women's mental health, gender-based violence and disclosure – three of the most core topics of importance to, and experienced by, women living with HIV, issues that are under-addressed and under-recognized, but fundamentally affect the experiences of women living with HIV in their most intimate lives. The first two papers are critical as they were led by women living with HIV giving the first-person's voice to the experience of mental health and violence, and both arose from the largest global survey of women living with HIV on sexual and reproductive health and rights priorities [9–11]. Building capacity for the community to be better heard within an academic context and further strengthening research to address gaps in our knowledge remain two critical priorities in order to build evidence-based guidance and recommendations. The systematic review of disclosure in the context of fear of violence [12] and the review of the needs of adolescent girls living with HIV [13] remind us that much remains to be achieved in the post-2015 era.

## Discussion

The past 20 years have seen tremendous progress in the area of sexual and reproductive health and rights. This is evidenced by an over 40% decrease in maternal mortality between 1990 and 2013, and a 58% increase in the use of modern contraceptive methods. The number of births to adolescents has also declined worldwide [14].

However, evidence shows the slow and uneven progress in various areas related to women and health, such as nutrition, sexual and reproductive health, HIV and other sexually transmitted infections and violence against women. Poor sexual and reproductive health outcomes represent one-third of the total global burden of disease for women between the ages of 15 and 49 years, with unsafe sex a major risk factor for death and disability among women and girls in low- and middle-income countries. In addition, worldwide, in 2013, 225 million women were estimated to have an unmet need for modern contraception.

When we look at the situation of women living with HIV in relation to sexual and reproductive health, the scenario remains bleak. In 2013, almost 60% of all new HIV infections among young people aged 15–24 years occurred among girls and young women. In low-income countries, tuberculosis is often linked to HIV infection and is among the leading causes of death of women of reproductive age and those aged 20–59 years.

Persistent obstacles in health systems to realizing the aims of the international declarations and conventions, including a lack of gender responsiveness with regard to sex-disaggregated data and gender analysis, result in health services that do not take into account the specific needs and determinants of women's health. Women, especially those living with HIV, continue to have inequitable access to good-quality health care services in many countries. Pockets of low health system coverage exist globally, and services in many rural areas and urban slums are often of low quality. Women living with HIV are confronted with multiple and intersecting forms of discrimination, which additionally contributes to the lack of good health services. Poor health service coverage is exacerbated by HIV status and gender-related barriers to access to prevention, treatment and care.

The papers in the Supplement aim to ensure that the sexual and reproductive health and human rights of women and girls living with HIV are addressed, with due attention accorded to the following critical dimensions:

1. placing human rights and gender equality at the centre of a comprehensive approach to health programming, in particular in relation to sexuality and sexual health;
2. ensuring health systems responsiveness to inequalities in access to health care and quality of care that often do not meet the needs of women living with HIV;
3. engaging and empowering women living with HIV in the development of policies and programmes that affect them; and
4. strengthening monitoring, evaluation and accountability procedures to provide good-quality data and ensuring remedial action against violations of health and human rights of women living with HIV.

## Conclusions: articulating a vision for the future

This Supplement includes contributions from a broad range of stakeholders on the complexity of issues related to sexual and reproductive health and human rights of women living with HIV. To realize this vision, the international development agenda in this regard should emphasize providing an enabling environment for women living with HIV to receive services that are based on principles of human rights and gender equality. Emphasis should also be placed on investing in integrated programmes interlinked with the different health-enhancing sectors, including, but not limited to, education and nutrition.
